# Supplementary material for: Economic, cultural, and social inequalities in potentially inappropriate medication: A nationwide survey- and register-based study in Denmark
Source: PLoS Med. 2024 Nov 20;21(11):e1004473. doi: 10.1371/journal.pmed.1004473 (PMC11578507; doi:10.1371/journal.pmed.1004473)
Supplement: S1 Table — (PDF) [file pmed.1004473.s001.pdf]

S1 Table: The RECORD statement

Checklist of items, extended from the STROBE statement, that should be reported in observational studies using routinely collected health data.

	Item No.	STROBE items	Location in manuscript where items are reported	RECORD items	Location in manuscript where items are reported
**Title and abstract**					
	1	(a) Indicate the study's design with a commonly used term in the title or the abstract (b) Provide in the abstract an informative and balanced summary of what was done and what was found	(a) Indicated in the title and in the abstract's methods section<br>(b) Indicated in the abstract	RECORD 1.1: The type of data used should be specified in the title or abstract. When possible, the name of the databases used should be included.<br><br>RECORD 1.2: If applicable, the geographic region and timeframe within which the study took place should be reported in the title or abstract.<br><br>RECORD 1.3: If linkage between databases was conducted for the study, this should be clearly stated in the title or abstract.	1.1. Indicated in the title and in the abstract.<br>1.2. Indicated in the title and in the abstract<br>1.3. Indicated in the abstract
**Introduction**					
Background rationale	2	Explain the scientific background and rationale for the investigation being reported	Explained in the introduction section		
Objectives	3	State specific objectives, including any prespecified hypotheses	Explained in the last paragraph of the introduction section		
**Methods**					

Study Design	4	Present key elements of study design early in the paper	Presented in the design section		
Setting	5	Describe the setting, locations, and relevant dates, including periods of recruitment, exposure, follow-up, and data collection	Described in the section 'Population and Setting'		
Participants	6	*(a) Cohort study* - Give the eligibility criteria, and the sources and methods of selection of participants. Describe methods of follow-up<br>*Case-control study* - Give the eligibility criteria, and the sources and methods of case ascertainment and control selection. Give the rationale for the choice of cases and controls<br>*Cross-sectional study* - Give the eligibility criteria, and the sources and methods of selection of participants<br><br>*(b) Cohort study* - For matched studies, give matching criteria and number of exposed and unexposed<br>*Case-control study* - For matched studies, give matching criteria and the number of controls per case	(a) Eligibility criteria, and sources and methods of selection of participants are described briefly in the two sections 'Population and Setting' and 'Data Sources' and elaborated elsewhere (referred in the design section [20]).	RECORD 6.1: The methods of study population selection (such as codes or algorithms used to identify subjects) should be listed in detail. If this is not possible, an explanation should be provided.<br><br>RECORD 6.2: Any validation studies of the codes or algorithms used to select the population should be referenced. If validation was conducted for this study and not published elsewhere, detailed methods and results should be provided.<br><br>RECORD 6.3: If the study involved linkage of databases, consider use of a flow diagram or other graphical display to demonstrate the data linkage process, including the number of individuals with linked data at each stage.	6.1. Described elsewhere. Referred to in the 'Design' section [20].<br>6.2. Not available<br>6.3. Full linkage was obtained. Missing data described in S2.
Variables	7	Clearly define all outcomes, exposures, predictors, potential confounders, and effect	Described in the methods section under 'outcome	RECORD 7.1: A complete list of codes and algorithms used to classify exposures, outcomes, confounders,	Provided in supplementary S3-S5.

		modifiers. Give diagnostic criteria, if applicable.	variables', 'exposure variables' and 'covariates'.	and effect modifiers should be provided. If these cannot be reported, an explanation should be provided.	
Data sources/ measurement	8	For each variable of interest, give sources of data and details of methods of assessment (measurement).<br>Describe comparability of assessment methods if there is more than one group	Described in the methods section under 'outcome variables', 'exposure variables' and 'covariates'.		
Bias	9	Describe any efforts to address potential sources of bias	Described in the second paragraph in the section 'statistical analyses'.		
Study size	10	Explain how the study size was arrived at	Described in the section 'population and setting'.<br>Response rates elaborated in S1.		
Quantitative variables	11	Explain how quantitative variables were handled in the analyses. If applicable, describe which groupings were chosen, and why	Described in the methods section under 'statistical analyses'. Groupings are described for each variable (please refer to item no. 7 'variables'.		
Statistical methods	12	(a) Describe all statistical methods, including those used to control for confounding<br>(b) Describe any methods used to examine subgroups and interactions	(a) Described in the section 'statistical analyses'.<br>(b) Described in the section 'statistical analyses'.		

		(c) Explain how missing data were addressed<br>(d) *Cohort study* - If applicable, explain how loss to follow-up was addressed<br>*Case-control study* - If applicable, explain how matching of cases and controls was addressed<br>*Cross-sectional study* - If applicable, describe analytical methods taking account of sampling strategy<br>(e) Describe any sensitivity analyses	(c) Described in the 'population and setting' section.<br>(d) Described elsewhere (referenced [20]).<br>(e) Described in the last paragraph of the section 'statistical analyses'.		
Data access and cleaning methods		..		RECORD 12.1: Authors should describe the extent to which the investigators had access to the database population used to create the study population.<br><br>RECORD 12.2: Authors should provide information on the data cleaning methods used in the study.	Both 12.1 and 12.2. are elaborated in the 'population and setting' section.
Linkage		..		RECORD 12.3: State whether the study included person-level, institutional-level, or other data linkage across two or more databases. The methods of linkage and methods of linkage quality evaluation should be provided.	Described in the 'data sources' section.
**Results**					
Participants	13	(a) Report the numbers of individuals at each stage of the study (*e.g.*, numbers potentially eligible, examined for eligibility,	Described in the 'population and setting' section and	RECORD 13.1: Describe in detail the selection of the persons included in the study (*i.e.,* study population selection) including filtering based on	Described in the 'population and setting' section.

		confirmed eligible, included in the study, completing follow-up, and analysed)<br>(b) Give reasons for non-participation at each stage.<br>(c) Consider use of a flow diagram	the 'descriptive data' section.	data quality, data availability and linkage. The selection of included persons can be described in the text and/or by means of the study flow diagram.	
Descriptive data	14	(a) Give characteristics of study participants (*e.g.*, demographic, clinical, social) and information on exposures and potential confounders<br>(b) Indicate the number of participants with missing data for each variable of interest<br>(c) *Cohort study* - summarise follow-up time (*e.g.*, average and total amount)	(a) Described in the 'descriptive data' section.<br>199-201; 204-9<br>(b) Indicated in supplementary table S2.		
Outcome data	15	*Cohort study* - Report numbers of outcome events or summary measures over time<br>*Case-control study* - Report numbers in each exposure category, or summary measures of exposure<br>*Cross-sectional study* - Report numbers of outcome events or summary measures	Reported in Table 2 and in the results section 'descriptive data'.		
Main results	16	(a) Give unadjusted estimates and, if applicable, confounder-adjusted estimates and their precision (e.g., 95% confidence interval). Make clear which	(a) Reported in Table 3 and in the results section 'PIM and adjusted prevalence		

		confounders were adjusted for and why they were included<br>(b) Report category boundaries when continuous variables were categorized<br>(c) If relevant, consider translating estimates of relative risk into absolute risk for a meaningful time period	proportion difference'.<br>(b) Reported in tables 2 and 3, and in S4 supplementary appendix.<br>(c) Not applicable		
Other analyses	17	Report other analyses done—e.g., analyses of subgroups and interactions, and sensitivity analyses	Reported in the results section 'mediation by long-term conditions'.		
**Discussion**					
Key results	18	Summarise key results with reference to study objectives	Reported in the discussion section in the first paragraph.		
Limitations	19	Discuss limitations of the study, taking into account sources of potential bias or imprecision. Discuss both direction and magnitude of any potential bias	Provided in the discussion section in the fifth paragraph.	RECORD 19.1: Discuss the implications of using data that were not created or collected to answer the specific research question(s). Include discussion of misclassification bias, unmeasured confounding, missing data, and changing eligibility over time, as they pertain to the study being reported.	Provided in the discussion section in the fifth paragraph.
Interpretation	20	Give a cautious overall interpretation of results considering objectives, limitations, multiplicity of analyses, results from similar studies, and other relevant evidence	Provided in the discussion section in the first, second and third paragraphs.		

Generalisability	21	Discuss the generalisability (external validity) of the study results	Provided in the discussion section in the fourth section.		
**Other Information**					
Funding	22	Give the source of funding and the role of the funders for the present study and, if applicable, for the original study on which the present article is based	Provided as a financial disclosure.		
Accessibility of protocol, raw data, and programming code				RECORD 22.1: Authors should provide information on how to access any supplemental information such as the study protocol, raw data, or programming code.	Provided in the 'design' section.[1]

*Reference: Benchimol EI, Smeeth L, Guttmann A, Harron K, Moher D, Petersen I, Sørensen HT, von Elm E, Langan SM, the RECORD Working Committee. The REporting of studies Conducted using Observational Routinely-collected health Data (RECORD) Statement. *PLoS Medicine* 2015; in press.

*Checklist is protected under Creative Commons Attribution (CC BY) license.

1. Paust A. Social Inequality in Medical Treatment. protocols.io. 2024. doi: dx.doi.org/10.17504/protocols.io.ewov19wy2lr2/v1.
