# Supplementary material for: Economic, cultural, and social inequalities in potentially inappropriate medication: A nationwide survey- and register-based study in Denmark
Source: PLoS Med. 2024 Nov 20;21(11):e1004473. doi: 10.1371/journal.pmed.1004473 (PMC11578507; doi:10.1371/journal.pmed.1004473)
Supplement: S2 Table — (PDF) [file pmed.1004473.s002.pdf]

S2 Table: Response rates in the Danish National Health Survey 2017

Characteristics	Invitations	Participants	Response rate (%)
	312,349	183,372	58.7
**Male**			
16-24 years	21,600	8,421	39.0
25-34 years	21,514	8,252	38.4
35-44 years	24,037	11,120	46.3
45-54 years	27,464	15,346	55.9
55-64 years	24,051	15,885	66.0
65-74 years	22,165	16,451	74.2
≥75 years	12,445	7,507	60.3
Total number	153,276	82,982	54.1
**Female**			
16-24 years	20,204	10,492	51.9
25-34 years	20,616	10,766	52.2
35-44 years	23,474	13,983	59.6
45-54 years	27,438	18,323	66.8
55-64 years	24,404	17,667	72.4
65-74 years	23,325	17,188	73.7
≥75 years	18,420	10,346	56.2
Total number	157,881	98,765	62.6
**Marital status**			
Married	148,087	100,168	67.6
Divorced	20,553	12,067	58.7
Widowed	35,012	20,655	59.0
Unmarried	108,697	50,482	46.4
**Ethnic origin**			
Danish	272,290	168,932	62.0
Western country	15,072	6,125	40.6
Non-western country	24,987	8,315	33.3
**Region**			
Capital Region of Denmark	112,644	59,267	52.6
Region Zealand	37,618	21,148	56.2
Region of Southern Denmark	64,173	43,231	67.4
Central Denmark Region	57,618	35,604	61.8
North Denmark Region	40,296	24,122	59.9
