# Supplementary material for: Economic, cultural, and social inequalities in potentially inappropriate medication: A nationwide survey- and register-based study in Denmark
Source: PLoS Med. 2024 Nov 20;21(11):e1004473. doi: 10.1371/journal.pmed.1004473 (PMC11578507; doi:10.1371/journal.pmed.1004473)
Supplement: S3 Table — (PDF) [file pmed.1004473.s003.pdf]

S3 Table: Missing data

Missing data					
	**Characteristics**	**Missing (%) N=170,495**	**PIM, Number**	**PIM, Mean**	**PIM, Standard deviation**
Economic capital	Wealth quintile categories	230 (0.3)	16	0.07	0.33
	Income quintile categories	81 (0.05)	0	0.00	0.00
Cultural capital	Immigration status	64 (0.04)	0	0.00	0.00
	Household education level	1,062 (0.62)	421	0.40	0.86
	Health education	4,166 (2.35)	802	0.19	0.62
Social capital	Social network	12,060 (6.79)	3135	0.26	0.68
	Cohabitation	13094 (7.68)	3059	0.23	0.64
	Social support	10,389 (5.85)	291	0.21	0.61
