# Supplementary material for: Economic, cultural, and social inequalities in potentially inappropriate medication: A nationwide survey- and register-based study in Denmark
Source: PLoS Med. 2024 Nov 20;21(11):e1004473. doi: 10.1371/journal.pmed.1004473 (PMC11578507; doi:10.1371/journal.pmed.1004473)
Supplement: S4 Table — (PDF) [file pmed.1004473.s004.pdf]

S4 Table: PIM criteria definitions according to the register-adapted STOPP/START criteria

ID	Description	A<br>ICD-10<br>*Atrial fibrillation*	B<br>ATC<br>*Vitamin K antagonists, direct thrombin inhibitors or factor Xa inhibitors*	Risk	Code
**START A1**	Start vitamin K antagonists or direct thrombin inhibitors or factor Xa inhibitors in the presence of chronic atrial fibrillation.	I48*	B01AA*, B01AE*, B01AF*	A if CHADVASc >=2	A & !B if CHADVASc >=2

ID	Description	A<br>ATC<br>*Vit K antagonists, direct thrombin inhibitors or factor Xa inhibitors*	B<br>ICD-10<br>*Coronary, cerebral or peripheral vascular disease*	C<br>Procedure codes<br>*Coronary implants (CABG or PCI)*	D<br>ATC<br>*Antiplatelet agents*	Risk	Code
**START A3**	Start antiplatelet therapy (acetylsalisylic acid or clopidogrel or prasugrel or ticagrelor) with a documented history of coronary, cerebral or peripheral vascular disease.	B01AA*, B01AE*, B01AF*	I20*, I21*, I22*, I23*, I24*, I25*, E10.5, E11.5, E14.5, I70.2, I73.9, I74.3, I63*, I64*, I65*, I66*, G45*	KFN*, KZFX01	B01AC*	!A & (B | C)	[!A & (B | C)] & !D

ID	Description	A<br>ICD-10<br>*Coronary, cerebral or peripheral vascular disease*	B<br>Procedure codes<br>*Coronary implants (CABG or PCI)*	C<br>ICD-10<br>*Chronic kidney disease*	D<br>ICD-10<br>*Rhabdomyolysis*	E<br>ATC<br>*Statins*	Risk	Code
**START A5**	Start statin therapy with a documented history of coronary, cerebral or peripheral vascular disease, unless the patient's status is end-of-life or age is > 85 years.	I20*, I21*, I22*, I23*, I24*, I25*, E10.5, E11.5, E14.5, I70.2, I73.9, I74.3, I63*, I64*, I65*, I66*, G45*	KFN*, KZFX01	N03*, N011*, N18*, N19*	M62.8, T79.6	C10AA*, C10B*	(A | B) & [!(C | [D within 6 months])]	{(A | B) & [!(C | [D within 6 months])]} & !E

ID	Description	A<br>ICD-10<br>*Heart failure*	B<br>ATC<br>*Angiotensin-converting-enzyme inhibitors or angiotensin II receptor blockers*	Risk	Code
**START A6**	Start ACE inhibitor with systolic heart failure and/or documented coronary artery disease.	I50*, I11.0, I13.0, I13.2	C09A*, C09B*, C09C*, C09D*	A	A & !B

ID	Description	A<br>ICD-10<br>*Ischaemic heart disease*	B<br>Procedure codes<br>*Coronary implants (CABG or PCI)*	C<br>ICD-10<br>*Heart failure*	D<br>ATC<br>*Beta blockers*	Risk	Code
**START A7**	Start beta-blocker with ischaemic heart disease.	I20*, I21*, I22*, I24*, I25*	KFN*, KZFX01	I50*, I11.0, I13.0, I13.2	C07*	(A | B) & !C	[(A | B) & !C] & !D

ID	Description	A<br>ICD-10<br>*Heart failure*	B<br>ATC<br>*Beta blockers (bisoprolol, nebivolol, metoprolol, or carvedilol)*	Risk	Code
**START A8**	Start appropriate beta-blocker (bisoprolol, nebivolol, metoprolol or carvedilol) with stable systolic heart failure	I50*, I11.0, I13.0, I13.2	C07AB02, C07AB07, C07AB12, C07AG02, C07BB02, C07BB07, C07BB12, C07BB52, C07CB02, C07FB02, C07FB07, C07FB12	A	A & !B

ID	Description	A<br>ICD-10<br>*Asthma or chronic obstructive pulmonary disease*	B<br>ATC<br>*Beta-2 agonists (inhalation) or antimuscarinic bronchodilators*	Risk	Code

**START B1**	Start regular inhaled b2 agonist or antimuscarinic bronchodilator (e.g. ipratropium, tiotropium) for mild to moderate asthma or COPD.	J40, J41*, J42, J43*, J44*, J45*, J46	R03AC*, R03AK04, R03AK05, R03AK06, R03AK07, R03AK08, R03AK09, R03AK10, R03AK11, R03AK12, R03AK13 ,R03AL*, R03BB*	A	A & !B

**ID**	**Description**	**A**<br>ICD-10<br>*Alzheimer's dementia or Lewy body dementia*	**B**<br>ATC<br>*Acetylcholinesterase inhibitors*	**Risk**	**Code**
**START C3**	Start acetylcholinesterase inhibitor (e.g. donepezil, rivastigmine, galantamine) for mild- moderate Alzheimer's dementia or Lewy Body dementia (rivastigmine).	G30*, F00*, G31.8	N06DA*	A	A & !B

<table>
<tr><th>ID</th><th>Description</th><th>A
ICD-10
Diabetes mellitus</th><th>B
ICD-10
Proteinuria</th><th>C
ICD-10
Diabetes mellitus with renal manifestations</th><th>D
ATC
Angiotensin-converting-enzyme inhibitors or angiotensin II receptor blockers</th><th>Risk</th><th>Code</th></tr>
<tr><td>START F1</td><td>Start ACE inhibitor or Angiotensin Receptor Blocker (if intolerant of ACE inhibitor) in diabetes with evidence of renal disease i.e. dipstick proteinuria or microalbuminuria (>30mg/24 hours) with or without serum biochemical renal impairment.</td><td>E10*, E11*, E12*, E13*, E14*</td><td>R80</td><td>E10.2, E11.2, E12.2, E13.2, E14.2, N08.3</td><td>C09A*, C09B*, C09C*, C09D*</td><td>(A & B (B later than A)) | C</td><td>((A & B (B later than A)) | C) & !D</td></tr>
</table>

<table>
<tr><th>ID</th><th>Description</th><th>A
ICD-10
Prostatism</th><th>B
ICD-10
Urinary retention</th><th>C
Procedure codes
Prostatectomy</th><th>D
ICD-10
Heart failure</th><th>E
ICD-10
Ortostatic hypotension or syncope</th><th>F
ATC
Alpha-1 blockers or 5-alpha-reductase inhibitors</th><th>Risk</th><th>Code</th></tr>
<tr><td>START G1</td><td>Start alpha-1 receptor blocker with symptomatic prostatism, where prostatectomy is not considered necessary.

Start 5-alpha reductase inhibitor with symptomatic prostatism, where prostatectomy is not considered necessary.</td><td>N40*</td><td>R33*</td><td>KKCC10, KKCC11, KKCC20, KKCC21, KKEC00, KKEC01, KKEC10, KKEC20</td><td>I50*, I11.0, I13.0, I13.2</td><td>I95.1, R55*</td><td>C02CA, G04CA*, G04CB*</td><td>(Male sex & [A | (B within 6 months)]) & !(C | D | [E within 6 months])</td><td>{(Male sex & [A | (B within 6 months)]) & !(C | D | [E within 6 months])} & !F</td></tr>
</table>

**ID**	**Description**	**A**<br>ATC<br>*Verapamil (and combinations) and diltiazem*	**B**<br>ATC<br>*Beta blockers*	**Risk**	**Code**
**STOPP B3**	Stop beta-blocker in combination with verapamil or diltiazem (risk of heart block)	C08DA01, C09BB10, C08DA51, C08DB01	C07*	All	A & B (in combination)

<table>
<tr><th>ID</th><th>Description</th><th>A
ICD-10
Bradycardia</th><th>B
ICD-10
AV block or other heart blocks</th><th>C
ATC
Beta blockers</th><th>Risk</th><th>Code</th></tr>
<tr><td>STOPP B4</td><td>Stop beta-blocker with bradycardia (<50/min), type II heart block or complete heart block (risk of complete heart block, asystole)</td><td>I49.5, R00.1</td><td>I44.1, I44.2, I44.3, I45.5, I45.9, Q24.6</td><td>C07*</td><td>(A within 6 months) | B</td><td>[(A within 6 months) | B] & C</td></tr>
</table>

**ID**	**Description**	**A**<br>ICD-10<br>*Supraventricular tachyarrhythmias*	**B**<br>ATC<br>*Beta blockers, digoxin, verapamil, or diltiazem*	**C**<br>ATC<br>*Amiodarone*	**Risk**	**Code**
**STOPP B5**	Stop amiodarone as first-line antiarrhythmic therapy in supraventricular tachyarrhythmias (higher risk of side-effects than beta-blockers, digoxin, verapamil or diltiazem)	I47.1, I48*	C01AA05, C07*, C08DA01, C08DB01	C01BD01	A & !B (within 3 years)	A & !B (within 3 years) & C

**ID**	**Description**	**A**<br>ICD-10	**B**<br>ICD-10	**C**<br>ICD-10	**D**<br>ATC	**E**<br>ATC	**Risk**	**Code**

		Hypertension	*Heart failure*	*Chronic kidney disease*	*Potassium-sparring agents, thiazides, beta blockers, calcium channel blockers, agents acting on the renin-angiotensin system*	*Loop diuretics*		
**STOPP B6**	Loop diuretic as first-line treatment for hypertension (safer, more effective alternatives available).	I10*, I15*	I50*, I11.0, I13.0, I13.2	N03*, N011*, N18*, N19*	C03D*, C03AA*, C07*, C08*, C09*	C03C*, C03EB*	A & ![B | C | (D within 3 years)]	(A & ![B | C | (D within 3 years)]) & E

**ID**	**Description**	**A**<br>ICD-10<br>*Heart failure*	**B**<br>ATC<br>*Phosphodiesterase-5 selective inhibitors*	**Risk**	**Code**
**STOPP B13**	Stop phosphodiesterase type-5 inhibitors (e.g. sildenafil, tadalafil, vardenafil) in severe heart failure characterized by hypotension i.e. systolic BP < 90 mmHg, or concurrent nitrate therapy for angina (risk of cardiovascular collapse)	I50*, I11.0, I13.0, I13.2	G04BE03, G04BE08, G04BE09, G04BE10	A	A & B

**ID**	**Description**	**A**<br>ICD-10<br>*Bleeding diathesis*	**B**<br>ICD-10<br>*Recent gastrointestinal bleeding*	**C**<br>ICD-10<br>*Cerebral hemorrhage*	**D**<br>ATC<br>*Antiplatelet agents, vitamin K antagonists, direct thrombin inhibitors, or factor Xa inhibitors*	**Risk**	**Code**
**STOPP C3**	Acetylsalisylic acid, clopidogrel, dipyridamole, vitamin K antagonists, direct thrombin inhibitors or factor Xa inhibitors with concurrent significant bleeding risk, i.e. uncontrolled severe hypertension, bleeding diathesis, recent non- trivial spontaneous bleeding) (high risk of bleeding).	D66*, D67*, D68.0*, D69.3	K92.0, K92.1, K92.2	I60*, I61*, I62*	B01AA*, B01AC*, B01AE*, B01AF*	A | ((B | C) within 6 months)	(A | ((B | C) within 6 months)) & D

**ID**	**Description**	**A**<br>ICD-10<br>*Ischaemic cerebrovascular disease*	**B**<br>ICD-10<br>*Acute coronary syndrome*	**C**<br>Procedure codes<br>*Coronary implants (CABG or PCI)*	**D**<br>ICD-10<br>*Carotid stenosis*	**E**<br>ATC<br>*Acetylsalicylic acid, carbasalate calcium, or combinations of antiplatelet agents*	**F**<br>ATC<br>*Clopidogrel*	**Risk**	**Code**
**STOPP C4**	Stop acetylsalisylic acid plus clopidogrel as secondary stroke prevention, unless the patient has a coronary stent(s) inserted in the previous 12 months or concurrent acute coronary syndrome or has a high grade symptomatic carotid arterial stenosis (no evidence of added benefit over clopidogrel monotherapy)	I63*, I64*, G45*	I20.0, I21*, I22*	KFN*, KZFX01	I65.2	B01AC06, B01AC08, B01AC56, B01AC30	B01AC04	A & {!([B | C] within 12 months) | !([D within 3 months] & [A within 3 months])}	[A & {!([B | C] within 12 months) | !([D within 3 months] & [A within 3 months])}] & (E & F in combination)

**ID**	**Description**	**A**<br>Procedure codes<br>*Coronary implants (CABG or PCI)*	**B**<br>ICD-10<br>*Acute coronary syndrome*	**C**<br>ATC<br>*Vitamin K antagonists, direct thrombin inhibitors, or factor Xa inhibitors*	**D**<br>ATC<br>*Antiplatelet agents*	**Risk**	**Code**
**STOPP C6**	Stop antiplatelet agents with vitamin K antagonist, direct thrombin inhibitor or factor Xa inhibitors in patients with stable coronary, cerebrovascular or peripheral arterial disease (No added benefit from dual therapy).	KFN*, KZFX01	I20.0, I21*, I22*	B01AA*, B01AE*, B01AF*	B01AC*	!(A | B) within 12 months	(![A | B] within 12 months) & (C & D (in combination))

**ID**	**Description**	**A**<br>ICD-10<br>*Deep venous thrombosis*	**B**<br>ICD-10<br>*History of pulmonary embolus*	**C**<br>ICD-10<br>*Atrial fibrillation*	**D**<br>Procedure codes<br>*Mechanical prosthetic heart valves*	**E**<br>ATC<br>*Vitamin K antagonists, direct thrombin inhibitors, or factor Xa inhibitors*	**Risk**	**Code**
**STOPP C8**	Stop vitamin K antagonist, direct thrombin inhibitor or factor Xa inhibitors for first deep venous thrombosis without continuing provoking risk factors (e.g. thrombophilia) for > 6 months, (no proven added benefit)	I80*	I26*	I48*	KFCA60, KFGE00, KFJF00, KFKD00, KFKD51, KFMD00, KFMD54	B01AA*, B01AE*, B01AF*	(A for the first time but more than 6 months ago) & !(B | C | D)	[(A for the first time but more than 6 months ago) & !(B | C | D)] & E

ID	Description	A<br>ICD-10<br>*Pulmonary embolus*	B<br>ICD-10<br>*History of deep venous thrombosis*	C<br>ICD-10<br>*Atrial fibrillation*	D<br>Procedure codes<br>*Mechanical prosthetic heart valves*	E<br>ATC<br>*Vitamin K antagonists, direct thrombin inhibitors, or factor Xa inhibitors*	Risk	Code
**STOPP C9**	Stop vitamin K antagonist, direct thrombin inhibitor or factor Xa inhibitors for first pulmonary embolus without continuing provoking risk factors (e.g. thrombophilia) for > 12 months (no proven added benefit)	I26*	I80*	I48*	KFCA60, KFGE00, KFJF00, KFKD00, KFKD51, KFMD00, KFMD54	B01AA*, B01AE*, B01AF*	(A for the first time but more than 12 months ago) & !(B | C | D)	[(A for the first time but more than 12 months ago) & !(B | C | D)] & E

ID	Description	A<br>ATC<br>*Vitamin K antagonists, direct thrombin inhibitors, or factor Xa inhibitors*	B<br>ATC<br>*Nonsteroidal anti-inflammatory drugs*	Risk	Code
**STOPP C10**	Stop NSAID and vitamin K antagonist, direct thrombin inhibitor or factor Xa inhibitors in combination (risk of major gastrointestinal bleeding).	B01AA*, B01AE*, B01AF*	M01A*, M01BA*, N02BA*	All	A & B (in combination)

ID	Description	A<br>ICD-10<br>*Dementia*	B<br>ICD-10<br>*Narrow angle glaucoma*	C<br>ICD-10<br>*Cardiac conduction abnormalities*	D<br>ICD-10<br>*Prostatism or urinary retention*	E<br>ATC<br>*Tricyclic antidepressants*	Risk	Code
**STOPP D1**	Stop tricyclic antidepressants (TCAs) with dementia, narrow angle glaucoma, cardiac conduction abnormalities, prostatism, or prior history of urinary retention (risk of worsening these conditions).	F00*, F01*, F02*, F03, G30*, G31.0, G31.1, G31.8	H40.2	I44*, I45*	N40*, R33*	N06AA*, N06CA01, N06CA02	A | B | C | (D within 6 months)	(A | B | C | (D within 6 months)) & E

ID	Description	A<br>ICD-10<br>*Prostatism or urinary retention*	B<br>ATC<br>*Chlorpromazine, clozapine, flupentixol, fluphenzine, pipotiazine, promazine, or zuclopenthixol*	Risk	Code
**STOPP D3**	Stop neuroleptics with moderate-marked antimuscarinic/anticholinergic effects (chlorpromazine, clozapine, flupenthixol, fluphenzine, pipothiazine, promazine, zuclopenthixol) with a history of prostatism or previous urinary retention (high risk of urinary retention).	N40*, R33*	N05AA01, N05AA03, N05AB02, N05AC04, N05AF01, N05AF05, N05AH02	(A within 6 months)	(A within 6 months) & B

ID	Description	A<br>ATC<br>*Benzodiazepines*	Risk	Code
**STOPP D5**	Stop benzodiazepines for ≥ 4 weeks (no indication for longer treatment; risk of prolonged sedation, confusion, impaired balance, falls, road traffic accidents; all benzodiazepines should be withdrawn gradually if taken for more than 4 weeks as there is a risk of causing a benzodiazepine withdrawal syndrome if stopped abruptly).	N03AE*, N05BA*, N05CD*, N05CF*	All	A, if more than 30 DDDs within 3 months

ID	Description	A<br>ICD-10<br>*Parkinsonism or Lewy Body dementia*	B<br>ATC<br>*Antipsychotics (excluding quetiapine, clozapine and lithium)*	Risk	Code
**STOPP D6**	Stop antipsychotics (i.e. other than quetiapine or clozapine) in those with parkinsonism or Lewy Body Disease (risk of severe extra-pyramidal symptoms)	G20, G21*, G23.1, G23.2, F02.3, G31.8	N05AA*, N05AB*, N05AC*, N05AD*, N05AE*, N05AF*, N05AG*, N05AH01, N05AH03, N05AH05, N05AH06, N05AK*, N05AL*, N05AX*	A	A & B

ID	Description	A<br>ICD-10<br>*Delirium*	B<br>ICD-10<br>*Dementia*	C<br>ATC<br>*Anticholinergic agents*	Risk	Code

<table>
<tr><td></td><td></td><td></td><td></td><td></td><td></td><td></td></tr>
<tr><td>STOPP D8</td><td>Stop anticholinergics/antimuscarinics in patients with delirium or dementia (risk of exacerbation of cognitive impairment).</td><td>F05*</td><td>F00*, F01*, F02*, F03*, G30*, G31.0, G31.1, G31.8</td><td>G04BD01, G04BD02, G04BD03, G04BD04, G04BD05, G04BD06, G04BD07, G04BD08, G04BD09, G04BD10, G04BD11, N03AF01, N03AF02, N05AA01, N05AA02, N05AA04, N05AB02, N05AB03, N05AB04, N05AC01, N05AC02, N05AF04, N05AG02, N05AH03, N05AH02, N05BB01, N05BB51, N06AA*, R01AX03, R03AC06, R03AL01, R03AL02, R03AL03, R03AL05, R03AL07, R03AL09, R03BB01, R03BB04, R03BB05, R03BB06, R03BB07, R06AA*, R06AB*, R06AC*, R06AD* , R06AE01, R06AE03, R06AE04, R06AE05, R06AE06, R06AE51, R06AE53, R06AE55, R06AX01, R06AX02, R06AX03, R06AX04, R06AX05, R06AX08, R06AX09, R06AX13, R06AX15, R06AX16, R06AX17,</td><td>(A within 6 months) | B</td><td>((A within 6 months) | B) & C</td></tr>
</table>

<table>
<tr><th>ID</th><th>Description</th><th>A
ICD-10
Dementia</th><th>B
ICD-10
Schizophrenic disorders or psychotic disorders</th><th>C
ICD-10
Sleeping disorders</th><th>D
ATC
Antipsychotics</th><th>Risk</th><th>Code</th></tr>
<tr><td>STOPP D9</td><td>Stop neuroleptic antipsychotic in patients with behavioral and psychological symptoms of dementia (BPSD) unless symptoms are severe and other non-pharmacological treatments have failed (increased risk of stroke).</td><td>F00*, F01*, F02*, F03*, G30*, G31.0, G31.1, G31.8</td><td>F20*, F25*, F29*</td><td>F51.0, G47.0</td><td>N05AA*, N05AB*, N05AC*, N05AD*, N05AE*, N05AF*, N05AG*, N05AH*, N05AK*, N05AL*, N05AX*</td><td>A & !B & !(C within 6 months)</td><td>(A & !B & !(C within 6 months)) & D</td></tr>
</table>

<table>
<tr><th>ID</th><th>Description</th><th>A
ICD-10
Dementia</th><th>B
ICD-10
Bradycardia</th><th>C
ICD-10
Heart block</th><th>D
ICD-10
Syncope</th><th>E
ATC
Beta blockers, digoxin, diltiazem, or verapamil</th><th>F
ATC
Acetylcholinesterase inhibitors</th><th>Risk</th><th>Code</th></tr>
<tr><td>STOPP D11</td><td>Stop acetylcholinesterase inhibitors with a known history of persistent bradycardia (< 60 beats/min.), heart block or recurrent unexplained syncope or concurrent treatment with drugs that reduce heart rate such as beta-blockers, digoxin, diltiazem, verapamil (risk of cardiac conduction failure, syncope and injury).</td><td>F00*, F01*, F02*, F03, G30*, G31.0, G31.1, G31.8</td><td>I49.5, R00.1</td><td>I44.1, I44.2, I44.3, I45.5, I45.9 , Q24.6</td><td>R55*</td><td>C01AA05, C07*, C08DA01, C08DA51, C08DB01, C09BB10</td><td>N06DA*</td><td>A & (B (within 6 months) | C | (D within 6 months) | E)</td><td>(A & (B (within 6 months) | C | (D within 6 months) | E)) & F (E and F in combination)</td></tr>
</table>

<table>
<tr><th>ID</th><th>Description</th><th>A
ATC
Antipsychotics</th><th>B
ATC
Phenothiazines</th><th>Risk</th><th>Code</th></tr>
<tr><td>STOPP D12</td><td>Stop phenothiazines as first-line treatment, since safer and more efficacious alternatives exist (phenothiazines are sedative, have significant anti-muscarinic toxicity in older people, with the exception of prochlorperazine for nausea/vomiting/vertigo, chlorpromazine for relief of persistent hiccoughs and levomepromazine as an anti-emetic in palliative care).</td><td>N05AE05, N05AH04, N05AL05, N05AX08, N05AX12, N05AX13</td><td>N05AA*, N05AB*, N05AC*, N05AX07</td><td>!(A within 3 years)</td><td>!(A within 3 years) & B</td></tr>
</table>

<table>
<tr><th>ID</th><th>Description</th><th>A
ATC
Adrenergic inhalants, glucocorticoid inhalants, or anticholinergic inhalants</th><th>B
ATC
Theophylline</th><th>Risk</th><th>Code</th></tr>
</table>

**STOPP G1**	Stop theophylline as monotherapy for COPD (safer, more effective alternative; risk of adverse effects due to narrow therapeutic index).	R03A*, R03BA*, R03BB*	R03DA04	!A	!A & B

ID	Description	A<br>ICD-10<br>*Narrow angle glaucoma*	B<br>ICD-10<br>*Prostatism or urinary retention*	C<br>ATC<br>*Antimuscarinic bronchodilators*	Risk	Code
**STOPP G3**	Stop antimuscarinic bronchodilators (e.g. ipratropium, tiotropium) with a history of narrow angle glaucoma (may exacerbate glaucoma) or bladder outflow obstruction (may cause urinary retention).	H40.2	N40*, R33*	R01AX03 , R03AL*, R03BB*	A | (B within 6 months)	(A | (B within 6 months)) & C

ID	Description	A<br>ATC<br>*Nonsteroidal anti-inflammatory drugs*	Risk	Code
**STOPP H6**	Stop long-term NSAID or colchicine (>3 months) for chronic treatment of gout where there is no contraindication to a xanthine-oxidase inhibitor (e.g. allopurinol, febuxostat) (xanthine-oxidase inhibitors are first choice prophylactic drugs in gout).	M01A*, M01BA*, N02BA*	All	A, if more than 90 DDDs within 6 months

ID	Description	A<br>ICD-10<br>*Coronary, cerebral or peripheral vascular disease*	B<br>Procedure codes<br>*Coronary implants (CABG or PCI)*	C<br>ATC<br>*COX 2 selective nonsteroidal anti-inflammatory drugs*	Risk	Code
**STOPP H7**	Stop COX-2 selective NSAIDs with concurrent cardiovascular disease (increased risk of myocardial infarction and stroke)	I20*, I21*, I22*, I23*, I24*, I25*, E10.5, E11.5, E14.5, I70.2, I73.9, I74.3, I63*, I64*, I65*, I66*, G45*	KFN*, KZFX01	M01AH*	A | B	(A | B) & C

ID	Description	A<br>ICD-10<br>*Dementia*	B<br>ICD-10<br>*Delirium*	C<br>ICD-10<br>*Narrow angle glaucoma*	D<br>ICD-10<br>*Prostatism and urinary retention*	E<br>ATC<br>*Bladder antimuscarinic agents*	Risk	Code
**STOPP I1**	Stop antimuscarinic drugs with dementia, or chronic cognitive impairment (risk of increased confusion, agitation) or narrow-angle glaucoma (risk of acute exacerbation of glaucoma), or chronic prostatism (risk of urinary retention).	F00*, F01*, F02*, F03*, G30*, G31.0, G31.1, G31.8,	F05*	H40.2	N40*, R33*	G04BD01, G04BD02, G04BD03, G04BD04, G04BD05, G04BD06, G04BD07, G04BD08, G04BD09, G04BD10, G04BD11	A | (B within 6 months) | C | (D within 6 months)	[A | (B within 6 months) | C | (D within 6 months)] & E

ID	Description	A<br>ICD-10<br>*Heart failure*	B<br>ATC<br>*Thiazolidenediones (e.g. rosiglitazone, pioglitazone) or combinations*	Risk	Code
**STOPP J2**	Stop thiazolidenediones (e.g. rosiglitazone, pioglitazone) in patients with heart failure (risk of exacerbation of heart failure)	I50*, I11.0, I13.0, I13.2	A10BD03, A10BD04, A10BD05, A10BD06, A10BD09, A10BD12, A10BG*	A	A & B

ID	Description	A<br>ICD-10<br>*History of breast cancer*	B<br>ICD-10<br>*History of venous thromboembolisms*	C<br>ATC<br>*Estrogens (or combinations)*	Risk	Code	Note
**STOPP J4**	Stop oestrogens with a history of breast cancer or venous thromboembolism (increased risk of recurrence).	C50*, D05*	I80*, I81*, I82*, I26*	G02BB*, G03AA*, G03AB*, G03CA01, G03CA06, G03CA07, G03CA09, G03CA53, G03CA57, G03CB*, G03CC*, G03EA*, G03EB*, G03F*, G03HB*, G03XC*, **G03CA03, G03CA04**	Female sex & (A | B)	(Female sex & (A | B)) & C	Estradiol (ATC: G03CA03) only, if whoadmvej = ”DEPPLA”|"GEL"|"TABFILM", and Estriol (ATC: G03CA04) only, if if whoadmvej = ”TAB”|"VAG"

ID	Description	A<br>ICD-10<br>*Hypogonadism*	B<br>ATC<br>*Androgens*	Risk	Code

**STOPP J6**	Stop androgens (male sex hormones) in the absence of primary or secondary hypogonadism (risk of androgen toxicity; no proven benefit outside of the hypogonadism indication).	E29.1, E89.5, Q98.0, Q98.1, Q98.2, Q98.4	G03B*	Male sex & !A	(Male sex & !A) & B

**ID**	**Description**	**A**<br>ICD-10<br>*Ortostatic hypotension or syncope*	**B**<br>ATC<br>*Vasodilator drugs (alpha- 1 blockers, calcium channel blockers, angiotensin-converting- enzyme inhibitors, or angiotensin II receptor blockers)*	**Risk**	**Code**
**STOPP K3**	Stop vasodilator drugs (e.g. alpha-1 receptor blockers, calcium channel blockers, long-acting nitrates, ACE inhibitors, angiotensin I receptor blockers,) with persistent postural hypotension<br>i.e. recurrent drop in systolic blood pressure ≥ 20mmHg (risk of syncope, falls)	I95.1, R55*	C01DA*, C02CA*, C02LE01, C07AG*, C07BG*, C07CG*, C08*, C09*, G04CA*	(A within 6 months)	(A within 6 months) & B

**ID**	**Description**	**A**<br>ATC<br>Selective beta-2-adrenoreceptor agonists	**B**<br>ATC<br>Adrenergics in combination with anticholinergics incl. triple combinations with corticosteroids	**C**<br>ATC<br>*Anticholinergic agents*	**D**<br>ATC<br>Antihistanines for systemic use	**E**<br>ATC<br>Carboxamide derivatives (antiepileptics)	**F**<br>ATC<br>Antipsychotics	**G**<br>Anxiolytics	**H**<br>ATC<br>Drugs for urinary frequency and incontinence	**I**<br>ATC<br>Non-selective monoamine reuptake inhibitors (antidepressants)	**Risk**	**Code**
**STOPP M**	Stop concomitant use of two or more drugs with antimuscarinic/anticholinergic properties	R03AC06	R03AL01, R03AL02, R03AL03, R03AL05, R03AL07, R03AL09	R03AX03, R03BB01, R03BB04, R03BB05, R03BB06, R03BB07	R06AA*, R06AB*, R06AC*, R06AD*, R06AE01, R06AE03, R06AE04, R06AE05, R06AE06, R06AE51, R06AE53, R06AE55, R06AX01, R06AX02, R06AX03, R06AX04, R06AX05, R06AX08, R06AX09, R06AX13, R06AX15, R06AX16, R06AX17, R06AX23, R06AX27, R06AX53, R06AX58	N03AF01, N03AF02	N05AA01, N05AA02, N05AA04, N05AB02, N05AB03, N05AB04, N05AC01, N05AC02, N05AF04, N05AG02, N05AH02, N05AH03	N05BB01, N05BB51	G04BD01, G04BD02, G04BD03, G04BD04, G04BD05, G04BD06, G04BD07, G04BD08, G04BD09, G04BD10, G04BD11	N06AA*	All	Two or more drugs from each of the subgroups concurrently (in combination)
