# Supplementary material for: Economic, cultural, and social inequalities in potentially inappropriate medication: A nationwide survey- and register-based study in Denmark
Source: PLoS Med. 2024 Nov 20;21(11):e1004473. doi: 10.1371/journal.pmed.1004473 (PMC11578507; doi:10.1371/journal.pmed.1004473)
Supplement: S5 Table — (PDF) [file pmed.1004473.s005.pdf]

S5 Table: Description of operationalization of capital forms

**Economic capital**		
Economic capital refers to material assets that are "immediately and directly convertible into money and may be institutionalized in the form of property rights".[1]		
**Variable**	**Operationalization**	**Description**
Wealth quintile categories	1: -47,303 EUR<br>2: 5,114 EUR<br>3: 36,270 EUR<br>4: 107,596 EUR<br>5: 426,100 EUR	Wealth was operationalized to encompass the complete portfolio information with respect to the value of bonds, stocks, cash in banks, real estate, mortgage loans, and the sum of other loans, excluding pension savings from assets due to inaccessibility to the holder.[2] The population was divided into quintiles based on their net value of assets and liabilities on Jan 1, 2017. The variable is calculated as the family-equivalent wealth, and the base population includes all individuals in families with at least one fully taxable person over 14 years of age.
Income quintile categories	1: 18,635 EUR<br>2: 27,426 EUR<br>3: 34,726 EUR<br>4: 43,591 EUR<br>5: 71,477 EUR	The population was divided into quintiles based on their family-equivalent disposable income on Jan 1, 2017. Disposable income was defined as the amount of money available for spending and saving after accounting for income taxes and interest expenses. The equivalized income allowed comparison between the household income of a single adult and that of a large family. Income was assumed to be perfectly redistributed within the family so that all family members received the same equivalized income. The OECD-modified scale was used for equalization. The first adult was assigned a weight of 1, other adults aged 14+ years were assigned a weight of 0.5 and children 0.3.
**Cultural capital**		
Cultural capital comprises the individual's cultural resources acknowledged in society, eg, etiquette preferences, linguistic styles, knowledge, or education attainment.[1]		
**Variable**	**Operationalization**	**Description**
Immigration status	Immigrant<br>Descendant<br>Danish	Immigration status was based on the assumption that an individual's understanding of, navigation in, and interaction with the healthcare system depend on the individual's level of familiarity and internalization of the culture within that system.[3] This may be particularly hard for immigrants and potentially also for descendants.<br>An immigrant was defined as an individual born abroad whose parents were both (or one of them if there was no available information on the other parent) foreign citizens or both born abroad. A descendant was defined as an individual born in Denmark whose parents (or one of them if there was no available information on the other parent) were either immigrants or descendants with foreign citizenship. If there was no available information on either of the parents, and the individual in question was a foreign citizen, this person was defined as a descendant.
Household education level	Primary and lower secondary<br>Upper secondary<br>Tertiary/bachelor<br>Master/doctoral	Education level was based on the highest attained education at household level. Each individual in the household was assigned the highest attained education level from the best-educated cohabitant. The rationale was that cultural capital is transmitted through socialization and is highly influenced by significant others.[4] For instance, two older women with short education who are married to sources with very different education levels are likely to possess very different levels of cultural capital.
Healthcare education	No<br>Yes	Having a healthcare-related education reflected the cultural capital arising from having field-specific knowledge, language, or merits that characterize the healthcare field. The variable was based on the formal classification of education programs by fields: dental studies, medicine, nursing and midwifery, medical diagnostics and treatment technology, therapy and rehabilitation, pharmacy, and traditional and complementary medicine and therapy. This operationalization did not differentiate between education levels but excluded everyone with the primary and lower secondary school as the highest attained education level because these levels are not field-specific.

**Social capital**		
Bourdieu's approach to social capital focuses on the actual and potential payoff from networks in the access to resources and opportunities.[4] The three measures of social capital were chosen based on the assumption that an individual's social capital is derived from both the household and the wider social network and that social support acts as an indicator for the actual and potential payoff from those networks.[5]		
**Variable**	**Operationalization**	**Description**
Social network	1 (infrequent)<br>2<br>3<br>4 (frequent)	The social network was operationalized as an index based on the Danish National Health Survey and was derived from the question "How often are you in contact with friends, acquaintances and family whom you do not live with? (contact means that you spend time together, talk on the phone, write to each other, etc.)" A complete response required choosing one of the following options "Daily or almost daily", "1 or 2 times a week", "1 or 2 times a month", "Less often than 1 time a month", or "Never" for each of the categories "Family members you do not live with", "Friends", "Colleagues or fellow students in your spare time", "Neighbors or residents in your local community". The final category "Online acquaintances (email, social media and similar)" was not included. Our operationalization allowed one missing item out of the four included categories. The combined score (4-20) was divided into four categories ranging from infrequent to frequent contact.
Cohabitation	Living alone or with child(ren)<br>Living with adult	Cohabitation was operationalized based on the Danish National Health Survey from the question "Do you live with others?" A complete response required responding "yes" or "no" to three statements: "I live with my spouse/partner/boyfriend/girlfriend", "I live with a child/children under the age of 16", and "I live with others aged 16 or over". We included individuals in "live with adult" if they were living with someone above the age of 16. If a child was below age 16, we considered such child incapable of providing support for medical treatment, and these individuals were categorized as living alone. Missing items were allowed if living with another adult could be confirmed or rejected from the incomplete response.
Social support	Never or almost never<br>Sometimes<br>Mostly<br>Always	Social support was operationalized based on the Danish National Health Survey from the question "Do you have someone to talk to if you have problems or need support?" Participants were asked to tick only one of the following responses: "Yes, always", "Yes, mostly", "Yes, sometimes", "No, never or almost never".

References

1. Bourdieu P. Forms of Capital. In: Richardson JG, ed. *Handbook of theory and research for the sociology of education*. Greenwood Press; 1986.
2. Boserup SH, Kopczuk W, Kreiner CT. Born with a Silver Spoon? Danish Evidence on Wealth Inequality in Childhood. *The Economic Journal*. 2018;128(612):F514-F544. doi:10.1111/ecoj.12496
3. Bourdieu P. *Language and Symbolic Power*. Harvard University Press. 1991.
4. Bourdieu P. The Forms of Capital. *Readings in Economic Sociology*. 2002:280-291. doi:10.1002/9780470755679.ch15
5. Pasgaard AA, Maehlisen MH, Overgaard C, Ejlskov L, Torp-Pedersen C, Boggild H. Social capital and frequent attenders in general practice: a register-based cohort study. *BMC Public Health*. Mar 2 2018;18(1):310. doi:10.1186/s12889-018-5230-2
