# Supplementary material for: Economic, cultural, and social inequalities in potentially inappropriate medication: A nationwide survey- and register-based study in Denmark
Source: PLoS Med. 2024 Nov 20;21(11):e1004473. doi: 10.1371/journal.pmed.1004473 (PMC11578507; doi:10.1371/journal.pmed.1004473)
Supplement: S6 Table — (PDF) [file pmed.1004473.s006.pdf]

S6 Table: Long-term conditions in the Danish Multimorbidity Index

Category	Disease group
Circulatory system	Hypertension
	Dyslipidemia
	Ischemic heart disease
	Atrial fibrillation
	Heart failure
	Peripheral artery occlusive disease
	Stroke
Endocrine system	Diabetes mellitus, types 1 and 2
	Thyroid disorder
	Gout
Pulmonary system and allergy	Chronic pulmonary disease
	Allergy
Gastrointestinal system	Ulcer/chronic gastritis
	Chronic liver disease
	Inflammatory bowel disease
	Diverticular disease of intestine
Urogenital system	Chronic kidney disease
	Prostate disorder
Musculoskeletal system	Connective tissue disorder
	Osteoporosis
	Painful condition
Hematological system	Anemia
	HIV/AIDS
Cancers	Cancer
Neurological system	Vision problem
	Hearing problem
	Migraine
	Epilepsy
	Parkinson’s disease
	Multiple sclerosis
	Neuropathy
Mental health conditions	Mood, stress-related, or anxiety disorder
	Psychological distress
	Alcohol problem
	Substance abuse
	Anorexia/bulimia
	Bipolar affective disorder
	Schizophrenia or schizoaffective disorder
	Dementia

Abbreviations: AIDS: acquired immunodeficiency syndrome; HIV: human immunodeficiency virus
