# Supplementary material for: Economic, cultural, and social inequalities in potentially inappropriate medication: A nationwide survey- and register-based study in Denmark
Source: PLoS Med. 2024 Nov 20;21(11):e1004473. doi: 10.1371/journal.pmed.1004473 (PMC11578507; doi:10.1371/journal.pmed.1004473)
Supplement: S1 Fig — (PDF) [file pmed.1004473.s007.pdf]

S1 Figure: Illustration of variables with Directed Acyclic Graph (DAG)

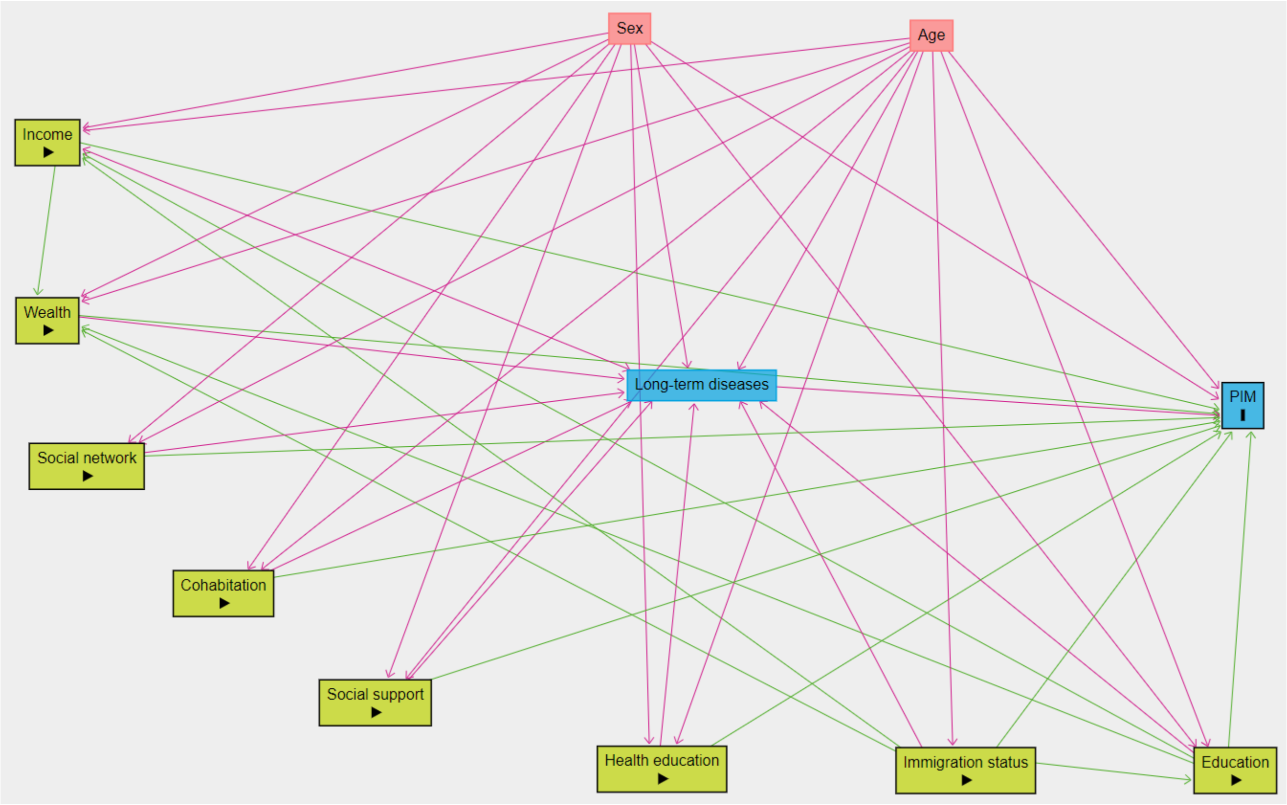

Illustration of the association between indicators of social position, long-term diseases, sex, age and potentitally inappropriate medications (Illustrated using DAGitty.com).
