# Supplementary material for: Economic, cultural, and social inequalities in potentially inappropriate medication: A nationwide survey- and register-based study in Denmark
Source: PLoS Med. 2024 Nov 20;21(11):e1004473. doi: 10.1371/journal.pmed.1004473 (PMC11578507; doi:10.1371/journal.pmed.1004473)
Supplement: Attachment — Submitted filename: paust.pdf [file pmed.1004473.s008.pdf]

This paper describes the association between social position and potentially inappropriate medication using routine datasets and an existing survey from Denmark.

I was asked for a statistical report and I interpret that to include all aspects of the design and conduct of the study.

Points of detail

Page 1 Perhaps worth adding Denmark in the title?

Page 3 I would have thought that the RECORD criteria (Benchimol et al., 2015) might perhaps have been more appropriate for a study based on several different routine datasets. The survey included here is not being reported on as a survey as far as I can see (when STROBE would have been relevant) but is just being used as another dataset.

Page 4 Strictly speaking the authors did not use the (four) quintiles in the regressions but divided into quintile categories or what lay people call fifths.

Page 5 What counts as a descendant here? Is this just first generation or could they have had a more remote ancestor born outside Denmark? The classification given in eTable 1 is quite different so how do these classifications match up?

Page 5 Why categorise age here? Categorising an essentially continuous variable wastes information (Altman and Royston, 2006; Royston et al., 2006) and leads to models which are often implausible as they predict the effect remaining flat within categories and then jumping to a new value at the category boundary. If, as we read later on this page, age is primarily just used as a covariate then some smooth function like splines or a low–degree polynomial would have been better.

Page 5 I am afraid I do not understand the sentence starting 'Before conducting the analysis' specifically how the last part 'which did not [...] analyzed separately' follows on from the rest.

Page 6 I do not think that the statement that they 'were subjected to 26252 PIMs' is correct. It is true that $26252 = 16761 + 5479 + 3144 + 609 + 259$ but that is the number of people with a PIM and does not take account of the multiple PIMs. So it should be $16761 + 2 \times 5479 + 3 \times 3144 + 4 \times 609 + 5 \times 259$ as a lower bound since I do not have the exact frequencies for 5+.

Page 6 It might be clearer to tell us how much social interaction the 33.6% had rather than saying it was below average.

Page 8 Perhaps show the relevant DAG in the methods?

Page 14, Table 1 Page 4 states that this is a representative sample. Is it really true that most Danes are women even given that men are fragile and die off at younger ages? In eT1 we see that the response rates for men and women differ especially for young men who, to nobody's surprise, do not answer surveys. The response rates may explain the sex ratio but it does rather undermine the claim to representativeness.

Page 14, Table 1 This combines STOPP and START. Should we see them separately?

Points of more substance

Is negative binomial the best model?

When I look at the figures though I am struck by the fact that the actual frequencies are rather different from what I might have expected. This suggests to me that an ordered categorical model might be more appropriate. Specifically a continuation ratio logistic model might give us an insight into (a) the odds of having any PIMs compared with none, and (b) the odds, conditional on having at least one PIM, of having more than one PIM. This might help to understand what the processes are which govern choosing a PIM.

actual	compare	crodds
151243	0:1+	0.15
16761	1:2+	0.36
5479	2:3+	0.42
3144	3:4+	0.22
609	4:5+	0.30
259		

Table 1: Odds

Assuming my calculations are correct the fact that the odds of having a PIM versus none are much lower than the odds of having more than one conditional on having one seems worth exploring in more detail.

Presentation of main results

The authors just compare the two most extreme fifths (on page 6, for example). This maximises the chances of being able to claim a statistically significant difference but it ignores most of the respondents. What we need is an overall test for each categorical variable with more than two levels. It would also help if the direction of differences was made explicit when results are described in the text on page 6. Saying that '[...] we found differences between the lowest and highest' is not really helpful.

Treating START and STOPP as the same

On page 7 the authors try to explain the findings of their economic variables. This raises a point which bothered me. They hint here that one might have expected different effects on STOPP and START. In that case why have they been amalgamated in the analysis?

Summary

A number of points for clarification and some points to think about in the analysis

Michael Dewey

References

D G Altman and P Royston. The cost of dichotomising continuous variables. *British Medical Journal*, 332:1080, 2006.

E I Benchimol, L Smeeth, A Guttmann, K Harron, D Moher, I Petersen, H Sørensen, E von Elm, S Langan, and RECORD working committee. The REporting of studies Conducted using Observational Routinely–collected health Data(RECORD) Statement. *PLOS Medicine*, 12(10), 2015.

P Royston, D G Altman, and W Sauerbrei. Dichotomizing continuous predictors in multiple regression: a bad idea. *Statistics in Medicine*, 25:127–141, 2006.
