# Supplementary material for: Economic, cultural, and social inequalities in potentially inappropriate medication: A nationwide survey- and register-based study in Denmark
Source: PLoS Med. 2024 Nov 20;21(11):e1004473. doi: 10.1371/journal.pmed.1004473 (PMC11578507; doi:10.1371/journal.pmed.1004473)
Supplement: Attachment — Submitted filename: Point-by-point review response_24_06_16.docx [file pmed.1004473.s009.docx]

**Point-by-point review response**

*Please note that page and line numbers refer to the clean document or the ‘simple markup’ view in the track-changes document.*

| **No.** | **Editorial comments** | **Response** |
| --- | --- | --- |
| **#1** | It took rather a long time to secure reviewers for your manuscript and we thank you for your patience. We agree with the reviewers and with the academic editor that your manuscript could be further strengthened and request that you respond to all comments in full. | Thank you for the comprehensive and thorough review of the manuscript. We have made every effort to incorporate the comments into the revised manuscript. Due to the extent of new analysis, we decided to remove the former model 3 (mediation analysis with mutual adjustment for other capital forms) from the manuscript. Should the editors or reviewers disagree with this decision, we can incorporate the model again. |
| **#2** | We agree that stratifying your analyses by sex would be a very valuable and we agree that investigating STOPP and START separately would also add additional insights and nuance. Please amend accordingly. | Thank you for this suggestion. We have stratified the results of the main analysis on both sex and the START/STOPP criteria, respectively. As they introduce a large number of results, we chose to put the sex-stratified analysis in supplementary material as we observed that male and female analysis are overall very similar. Additionally, as explained in the description of the capital forms (eTable 4), the data for income, wealth, and educational attainment is reported at a household level. Therefore, conducting sex-stratified analyses for these variables is less meaningful. We have updated the methods in the statistical analyses section (Page 5, lines 166- 183) and elaborated on the results (page 7, lines 228-255). |
| **#3** | We agree with the statistical reviewer that reporting your study according to RECORD would be appropriate. Please see here for further details <https://www.equator-network.org/reporting-guidelines/record/>. When completing the checklist, please use section and paragraph numbers, rather than page numbers as these often change in event of publication. | Thanks for the advice. We have edited the manuscript in accordance with the RECORD reporting guidelines and mentioned the criteria in the design section (page 4, line 106). Please see the updated population and setting in the methods section (page 4, lines 109-119), added a data sharing statement (page 10, lines 347-351) and commented on the generalizability of the study (page 9, lines 313-315 and 329-331) |
| **#4** | For all observational studies we request that in the manuscript text, you please indicate: (1) the specific hypotheses you intended to test, (2) the analytical methods by which you planned to test them, (3) the analyses you actually performed, and (4) when reported analyses differ from those that were planned, transparent explanations for differences that affect the reliability of the study's results. If a reported analysis was performed based on an interesting but unanticipated pattern in the data, please be clear that the analysis was data-driven. | Thank you for this suggestion. We have added our hypothesis to the introduction section as follows: “*We hypothesized that all forms of capital would be inversely associated with risk of receiving PIM and that multimorbidity would mediate some of the association.”*(page 3, lines (101-102)  Moreover, we have specified that our initial intention was to investigate the association between indicators of social position with PIM  Please refer to #6 for more comments on the study protocol. |
| **#5** | Did your study have a prospective protocol or analysis plan? Please state this (either way) early in the Methods section. | We have added a comment to the design section in the manuscript as follows: “*The study was based on a prospective protocol*.” (Page 4, line 107) Please see comment #6. |
| **#6** | If a prospective analysis plan (from your funding proposal, IRB or other ethics committee submission, study protocol, or other planning document written before analyzing the data) was used in designing the study, please include the relevant prospectively written document with your revised manuscript as a Supporting Information file to be published alongside your study, and cite it in the Methods section. A legend for this file should be included at the end of your manuscript. | This study was part of a PhD project that included four separate studies, and thus, each study is described very briefly in the official protocol. However, the study was described in more detail in an internal document prior to data analysis. This document is, in our opinion, unfit for publication, but we have submitted it as supporting material for your information. |
| **#7** | If no such document exists, please make sure that the Methods section transparently describes when analyses were planned, and when/why any data-driven changes to analyses took place. | We have added some comments for this in the statistical analysis section as follows “*A brief study protocol informed the study, including hypothesis, main analysis, and specifications for variables, but amendments were made after internal and external review, adding posthoc analysis to the study. These include the mediation analysis (from the inclusion of DAGs based on internal review) and stratified analysis (suggested in external review). In addition, the study was planned as a 1-year follow-up study but was conducted as a cross-sectional study based on prospectively collected data due to the risk of time-dependent bias*.” (Page 6, lines 190-195). |
| **#8** | In either case, changes in the analysis -- including those made in response to peer review comments-- should be identified as such in the Methods section of the paper, with rationale. | Thank you for this suggestion. We have added some comments on the study protocol and the post-hoc analysis in the manuscripts. Please see #7 |

| **Comments** | **Reviewer #1** | **Response** |
| --- | --- | --- |
| **#9** | This paper describes the association between social position and potentially inappropriate medication using routine datasets and an existing survey from Denmark. I was asked for a statistical report and I interpret that to include all aspects of the design and conduct of the study. | Thank you for this comment. We sincerely appreciate your thoughtful review of our paper. |
| **#10** | **Points of detail** |  |
| **#11** | Page 1 Perhaps worth adding Denmark in the title? | We have added ‘Denmark’ to the title |
| **#12** | Page 3 I would have thought that the RECORD criteria (Benchimol et al., 2015) might perhaps have been more appropriate for a study based on several different routine datasets. The survey included here is not being reported on as a survey as far as I can see (when STROBE would have been relevant) but is just being used as another dataset. | We agree that the RECORD criteria are appropriate for the study and, hence, have edited the manuscript to report according to the criteria. Specifically, we mention the criteria in the design section (page 4, line 106). Please see the updated population and setting in the methods section (page 4, lines 109-119), added a data sharing statement (page 10, lines 347-351) and commented on the generalizability of the study (page 9, lines 313-315 and 329-331) |
| **#13** | Page 4 Strictly speaking the authors did not use the (four) quintiles in the regressions but divided into quintile categories or what lay people call fifths. | Thank you - we agree. We have changed the wording to ‘quintile categories’ throughout the manuscript. |
| **#14** | Page 5 What counts as a descendant here? Is this just first generation or could they have had a more remote ancestor born outside Denmark? The classification given in eTable 1 is quite different so how do these classifications match up? | Thank you for this point. We understand the confusion about ethnic origin and immigration status. In eTable 1, we report the response rates as they are collected in the Danish National Health Survey (where the variable on ethnic origin is used to ensure representativeness in the respondents and to analyse the response rate), but in our study, we chose to use immigration status in our operationalization, because we theorized that an individual’s understanding of, navigation in, and interaction with the healthcare system depend on the individual’s level of familiarity and internalization of the culture within that system. We have specified that the full description of the operationalization is found in eTable 4. (Page 15, lines 486) |
| **#15** | Page 5 Why categorise age here? Categorising an essentially continuous variable wastes information (Altman and Royston, 2006; Royston et al., 2006) and leads to models which are often implausible as they predict the effect remaining at within categories and then jumping to a new value at the category boundary. If, as we read later on this page, age is primarily just used as a covariate then some smooth function like splines or a low{degree polynomial would have been better. | Thank you for this suggestion. We agree that age could be operationalized better and have changes the categorical variables to a restricted cubic splines function, using 5 knots. This has been added to the description of covariates in the methods section (page 5, lines 161-162). |
| **#16** | Page 5 I am afraid I do not understand the sentence starting `Before conducting the analysis' specifically how the last part `which did not [. . . ] analyzed separately' follows on from the rest. | We recognize that this was an insufficient description. We have added more details so that it now reads: “*Before conducting the analysis, we checked for possible collinearity between exposure variables and only saw substantial collinearity between indicators within the same capital form, e.g. wealth and income. Yet, variables from the same capital form did not enter the same analysis as all eight indicators were analyzed separately*.” (Page 6, lines 181-184). |
| **#17** | Page 6 I do not think that the statement that they `were subjected to 26252 PIMs' is correct. It is true that 26252 = 16761+5479+3144+609+259 but that is the number of people with a PIM and does not take account of the multiple PIMs. So it should be 16761+2x5479+3x3144+4x609+5x259 as a lower bound since I do not have the exact frequencies for 5+. | We fully agree – this has been corrected in the manuscript to write “*Overall, 26,252 individuals in the study population were subjected to 39,970 PIMs at the time of the data collection*.” (Page 6, lines 205-208) |
| **#18** | Page 6 It might be clearer to tell us how much social interaction the 33.6% had rather than saying it was below average. | As described in eTable 4, the social network was operationalized as an index based on the Danish National Health Survey and was derived from the question “How often are you in contact with friends, acquaintances and family whom you do not live with? (contact means that you spend time together, talk on the phone, write to each other, etc.)” A complete response required choosing one of the following options “Daily or almost daily”, “1 or 2 times a week”, “1 or 2 times a month”, “Less often than 1 time a month”, or “Never” for each of the categories “Family members you do not live with”, “Friends”, “Colleagues or fellow students in your spare time”, “Neighbors or residents in your local community”. As they are all combined in one measure, the only meaningful way to report it is relative to the entire study population's response. |
| **#19** | Page 8 Perhaps show the relevant DAG in the methods? | Thank you for this suggestion. We have added the DAG to the supplementary material. |
| **#20** | Page 14, Table 1 Page 4 states that this is a representative sample. Is it really true that most Danes are women even given that men are fragile and die off at younger ages? In eT1 we see that the response rates for men and women differ especially for young men who, to nobody's surprise, do not answer surveys. The response rates may explain the sex ratio but it does rather undermine the claim to representativeness. | Thank you for this comment. We have elaborated on the sampling in the population and setting section in the methods as follows:  *“The invited population were selected by Statistics Denmark as a random sample of the adult Danish population for the survey year based on an algorithm to ensure geographic and demographic representativeness.”* (Page 4, lines 110-113)  Of course, questionnaires are always subjected to selection problems, but we have tried to minimize these by considering age, sex and long-term conditions in the analysis. |
| **#21** | Page 14, Table 1 This combines STOPP and START. Should we see them separately? | We agree. This has now been edited in the manuscript in the methods section (page 5, lines 177-178) and the results section (page 7, lines 242-255). |
| **#22** | **Points of more substance** |  |
| **#23** | **Is negative binomial the best model?**  When I look at the figures though I am struck by the fact that the actual frequencies are rather different from what I might have expected. This suggests to me that an ordered categorical model might be more appropriate. Specifically a continuation ratio logistic model might give us an insight into (a) the odds of having any PIMs compared with none, and (b) the odds, conditional on having at least one PIM, of having more than one PIM. This might help to understand what the processes are which govern choosing a PIM.  actual compare crodds  151243 0:1+ 0.15  16761 1:2+ 0.36  5479 2:3+ 0.42  3144 3:4+ 0.22  609 4:5+ 0.30  259  Table 1: Odds  Assuming my calculations are correct the fact that the odds of having a PIM versus none are much lower than the odds of having more than one conditional on having one seems worth exploring in more detail. | Thank you for this suggestion, which we appreciate was very well-thought through.  We have made substantial edits to our analysis, so that we now divide into “any PIM” using logistic regression, and “number of PIMs” conditioned on at least one. It appears that the odds of having PIM is much greater for the first PIM compared to the following PIMs. Please see the methods section (page 5, lines 174-176) and the results section for edited analysis (model 1a & 1b, page 7, lines 232-241). |
| **#24** | **Presentation of main results**  The authors just compare the two most extreme fifths (on page 6, for example). This maximises the chances of being able to claim a statistically significant difference but it ignores most of the respondents. What we need is an overall test for each categorical variable with more than two levels. It would also help if the direction of differences was made explicit when results are described in the text on page 6. Saying that `[. . . ] we found differences between the lowest and highest' is not really helpful. | We believe that the presentation of our results is very transparent in the effect sizes of the associations. We do however agree that the presentation could be more precise and have specified that a dose-response association was observed for most of the analyses (page 7, lines 232-242) |
| **#25** | **Treating START and STOPP as the same**  On page 7 the authors try to explain the findings of their economic variables. This raises a point which bothered me. They hint here that one might have expected different effects on STOPP and START. In that case why have they been amalgamated in the analysis? | Thank you for this comment. We have stratified our analysis on START and STOPP to address this issue and updated the methods section (page 5, lines 177-178) and the results section (page 7, lines 242-255). |
| **#26** | **Summary**  A number of points for clarification and some points to think about in the analysis | We sincerely thank you for your thorough review and helpful comments. |

| **Comments** | **Reviewer #2** | **Response** |
| --- | --- | --- |
| **#27** | This is an interesting manuscript, aiming to divulge more of the hard to define concept social position and its relation to health inequalities. The article looks at potentially inappropriate medication (PIM) and social inequality. By using Bourdieu´s capital theory the authors are including not only economic and cultural capital but also social capital. By using individual answers from a household survey in combination with register data they are able to analyse the association between PIM and social inequality on an individual level. | Thank you for your interest and for your very helpful suggestions. |
| **#28** | They can confirm previous findings regarding social inequality expressed by economic and cultural capital and these findings are also detected regarding social capital, however weaker. |  |
| **#29** | Despite this, I have a couple of remarks regarding the manuscript: My main objection is that the results are not reported separately for men and women. Women utilize more health care and consume more medicine and in my mind, this is a fundamental reason to make a gender division. | Thank you for this suggestion. We agree that these analyses are very interesting, and thus, we have added the stratified analysis to the study. However, due to the extent of the analysis and as the results are not radically different between men and women, we have addressed this in the statistical analyses section (page 5, line 178) and commented on this finding in the Results section (page 7, lines 251-256) but we provide the full analysis as supplementary materials in eFigure 2. |
| **#30** | Minor objections: In the introduction where the authors outline social class and there will to analyse according to Bourdieu´s work no direct reference is made to his work, only indirect references. Although this reference is made in the supplement, it is appropriate to have it in the main text. | We have added the direct references to the introduction (page 3, lines 91-93). Thank you for highlighting this. |
| **#31** | In the first section of the results section (descriptive) a textual briefing of table 2 is made. Unfortunately, the order in the briefing and the table are not equivalent. Better if the orders not are different. | Thank you, we agree. We have edited this section accordingly. |
| **#32** | In table 3, please check the count figures for wealth quintiles. It looks as if the order is wrong. | We appreciate your concern. We have double checked this and the count figures appear in the right order. We expect that they appear suspicious because the number of PIMs are higher in the higher wealth groups? In that case, it is because age is such a strong confounder, with increased wealth in higher age, but at the same time increasing risk of PIM with increased age. |
| **#33** | The authors have analysed each variable by its own but it would have been interesting to see some example of interaction, for example can high social capital compensate low economic capital. | Thank you for this suggestion. We have investigated interactions between the indicators of social position but did not recognize any patterns to prompt the inclusion of an interaction analysis. Since the article and supplementary materials are already extensive, we have decided not to include the interaction analysis in the manuscript, but it would be interesting to investigate in-depth in a future study. We have added a comment in the discussion section as follows “*However, further investigations are needed to explore other indicators of treatment quality and the interplay between capitals to understand the underlying differences in the medical treatment of various social groups*.” (page 10, lines 334-336) |
| **#34** | All in all, I find this interesting not only for me but for a wider audience if corrections are made. | Thank you again for your review and your suggestions. We hope that the reviewer agrees with the edits made to the manuscript. |

|  | **Reviewer #3** | **Response** |
| --- | --- | --- |
| **#35** | Thank you for the opportunity to review the manuscript. This is an interesting and novel study investigating the association between social position and quality of medication use. The research question and statistical analysis are generally well-defined and straight forward. | Thank you very much for a constructive review – we hope that we have accommodated sufficiently and responded satisfactory to the critique. |
| **#36** | The main limitation is that the theoretical framework is not sufficiently integrated and that the outcome and some analytical decisions should be further problematized/discussed. Below, I list some issues that in my view, would improve the paper. | Thank you for this comment, which certainly made us reflect on our application of capital theory in the study. Please see our response #38. |
|  | **Main comments:** |  |
| **#37** | I am supportive of the authors decision to use more indicators than education/income/occupation to assess social position. However, I am a bit skeptical about the theoretical connection to Bourdieu's capital terminology. | We kindly refer to the supplementary material which now includes more justification on the use of theory in the operationalization of the analyses (eTable 4) |
| **#38** | Although I am far from an expert on Bourdieu's work, I have some recommendation to potentially bridge the gap between the analysis and the theoretical discussion: First, the mechanisms underlying the associations as presented in the Discussion should be expanded. Currently, the presented mechanisms are largely materialistic and immediate in nature. In my mind, Bourdieu's work is more concentrated on how social stratification is transferred over time periods/generations and how the capital is used to distinguish/differentiate oneself in relation to other people. It is a bit hard to see how this relates to inappropriate medication use. Second, including healthcare-related education as a form of Cultural capital fits well with the study at hand but is more unclear in relation to the term cultural capital in general, and should be better justified. | Thank you for your comments. We believe that Bourdieu’s work has various applications. In our study, we used the concept of capital to identify important indicators of social position for examining social inequality in medical treatment. However, we acknowledge that the theoretical concept encompasses much more, as mentioned by the reviewer, which was challenging to address in this specific study. Nevertheless, we added further discussion on the operationalization (e.g., the transfer of the value of capital within households) in the supplementary materials (See eTable 4). This is referred to in the methods section (page 4, line 142) and has been added to table 1 as well. In eTable 4, you can also find more details on how healthcare-related education fits into the theoretical framework. To further embed the study in a theoretical discussion, we included additional comments in the discussion section as follows: ”*Drawing from Bourdieu's work, social inequality is perpetuated through social interactions and power dynamics, underscoring the complex and interconnected nature of the mechanisms behind unequal access to medical treatment*.” (page 8-9, lines 293-295). |
| **#39** | I have some comments relating to the selected outcome: a) Please provide a reference to the STOPP/START criteria, also indicating which version of STOPP/START you used. b) The STOPP/START criteria were developed specifically for older adults, because some medications that are appropriate in other age groups are inappropriate in older adults (e.g. NSAIDs). Hence, you will need to motivate the use of these criteria in other age groups. | Thank you for your suggestions. We have added some more details to the description of the START/STOPP criteria and added a reference to the O’Mahony study (page 4, lines 132-138).  We build on extensive work by Ribe et al 2021, who have operationalized the STOPP/START criteria to fit Danish national registers. Their work entails a comprehensive process to select and fit the relevant criteria to a Danish context and a broader population. They have considered several measures of the study population in selecting the criteria to allow them to fit the general population including those of a younger age. This has also been added to the methods section (page 5, line 133). |
| **#40** | c) The START criteria are often not included in studies about medication quality, I would be interested to see the results for STOPP and START separately, also as there might be slightly different process generating inequality for the two different domains. | We have separated the analysis to accommodate the request and found some interesting differences. Thank you for suggesting the stratified analysis. |
| **#41** | d) It is clearly stated that the PIMs were calculated as a one-day point prevalence (which is a good decision especially for drug-drug interactions). However, I can not find information on the method used to calculate the one-day point prevalence, how long was the look-back period, was duration calculated using the DDD-method etc.? | We now refer more explicitly to Ribe et al. 2021 and Prior et al. 2023 for more information on the operationalisation of the STOPP/START variables in the Methods Section, xx as follows: . *“The operationalization of PIM is described in eTable 3, and more detailed descriptions of the methods can be found elsewhere.24,25”* (page 4, lines 136-137) For your convenience, we have copied some of the explanations from Ribe et al 2021 in a quote below: “*After redeeming a prescription of a drug in any of the PIM categories, individuals were considered continuous ‘users’ of that drug for a period of time corresponding to the number of redeemed defined daily doses (DDD), that is, the assumed average maintenance dose per day for a drug used for its main indication in adults, plus a grace period of 25% to allow for some leeway when redeeming prescriptions.28 This period of use was extended each time a user redeemed a new prescription. For the PIM criteria defined by two concurrent treatments, some individuals were excluded during quarantine periods in-between treatments, as we could not distinguish between overlapping treatments and treatment shifts before the first prescription expired (see details in Supplemental methods, online supplemental material 1*).” |
| **#42** | The analytical decision to conduct mediation analysis to address long-term conditions should be better supported. I generally agree with the decision, but I think it could be more clearly stated what it is and what the benefits are. | We appreciate your suggestion and have added our hypothesis about mediation in the introduction as follows:  “*We hypothesized that all forms of capital would be inversely associated with risk of receiving PIM and that multimorbidity would mediate some of the association*.” (page 3, lines 101-102)  Moreover, we believe that it was already sufficiently addressed in the background section from the following phrase “*An inverse association between social position and PIM has already been established.8,9 Yet, when investigating the connection, studies tend to ignore the differential disease risk between social groups, which mediates much of the association between social position and PIM.9*” (page 3, lines 81-84)  However, to further strengthen the choice of mediation analysis, we added the directed acyclic graph to the supplementary (eFigure 1) and emphasized the arguments for mediation analysis in the methods section on statistical analysis (page 4, lines 142-143) |
|  | **Minor comments:** |  |
| **#43** | Does reference #5 really support the authors statement? | We believe that it does. For instance, the study by Mackenbach et al. 2019 states “*Income inequality has gone up [29], and this has been associated with rising mortality, but more so among the low than the high educated, suggesting an inequalities-widening effect*.” Moreover, the study found that it is particularly true in a Nordic setting: “*The worst performers, both among men and among women, are three Nordic countries: Finland, Norway and Denmark. We have demonstrated previously in cross-sectional analyses that, unlike what one would expect on the basis of their universal and generous welfare states, these countries do not have smaller health inequalities than other European countries [27]. This new analysis shows that over the past decades, these countries have also experienced relatively unfavourable trends in inequalities in mortality.*”. |
| **#44** | The category 'living alone or with child(ren)' is unclear and should be re-named. | We agree and have edited the variable labels of the cohabitation variable to be “living with adult(s) (≥age 16)” and “not living with adult(s) (≥age 16)” |
| **#45** | I recommend removing "strongly" in the first sentence of the Discussion (Row 226) | This is a good point – we have edited according to the suggestion. |

|  | **Academic Editor** | **Response** |
| --- | --- | --- |
| **#46** | Overall, I think it is quite well done but I think opportunities to make it quite strong. Happy to take a look after reviews are in or after authors respond to them. | Thank you for your positive words and your thorough review. |
| **#47** | This is a well-written article using a robust dataset. Epidemiological analyses are well-thought out and theoretically informed. A few suggestions for improvements to make analyses more robust. |  |
| **#48** | -Consider differences between medications that should have been started but weren’t vs. not stopping a necessary medication. Particularly in terms of socioeconomic and racial disparities, access is substantial driver and will mostly affect whether necessary medication not started. I think I would be more interested in these separately rather than an aggregate measure as I think the mechanisms are likely quite different (quality itself is a broad construct and has many dimensions). Consider reporting each separately rather in# aggregate. | This is a really helpful suggestion. We have now performed the stratified analysis on START and STOPP PIM to investigate if there were any differences between the two types and added this to the study and this has been reported in the Methods and Results as outlined in our response to Comment #2 |
| **#49** | -See Levesque et al framework on access. I think this can complement existing theoretical foundation and perhaps fine tune analysis/conceptualization. [https://equityhealthj.biomedcentral.com/articles/ 10.1186/1475-9276-12-18](https://equityhealthj.biomedcentral.com/articles/10.1186/1475-9276-12-18). | Thank you for this suggestion. We agree that the framework is very relevant to this study and, thus, we have commented on this in the discussion section (page 9, lines 302-305) |
| **#50** | -I am confused about adjusted risk differences presented. e.g., for wealth, it seems like mean increases as wealth goes goes up, but this is opposite of adjusted risk difference. Is it true the adjustment fully reversed direction of relationship? | Thank you for this helpful comment. Yes indeed – age is a very strong confounder in this regard. And since these measures are crude, they do not consider the strong association between higher age and accumulated wealth on one hand and old age and increased risk of diseases and PIM on the other. Hence, we have not made any amendments to the manuscript. |
| **#51** | -I think it would be quite interesting to also consider interactions between different types of capital. E.g., lack of social capital may be particularly exacerbated in the presence of lack of economic capital. High economic capital may counteract impact of low social capital etc. | We agree that this could be very interesting. However, we believe that the number of capital indicators used in this study complicates the interaction sub-analyses, as this would be an additional 8x8 analyses. We believe that it is an excellent suggestion for future research and, thus, this has been added to the discussion section as follows:  “*However, further investigations are needed to explore other indicators of treatment quality and the interplay between capitals to understand the underlying differences in the medical treatment of various social groups*.”. (page 10, lines 334-336) |
| **#52** | -I think there is also an element of who your contacts are. This may be captured in the interaction between social and economic capital (e.g., are you connect to well off and well connected individuals?). I imagine the data doesn’t actually capture this directly, but this could provide useful inferences. | Agreed. This is also why we used measures on a household level to capture economic capital. This would be interesting to explore in more detail, but we would argue that other study designs would be more fit to explore this interaction (we hope to follow up on this idea in another study). Please refer to more details in comment #53. |
| **#53** | -In truth, there may be complex interactions between the different type of capital that essentially identify different subgroups phenotypes. Although complicated to do, using latent class analysis with the different measures of social position to identify these phenotypes and then using this as the exposure would be quite interesting. Could think of other methods for segmentation as well. | Latent class analysis would be a very interesting approach to this topic. We will investigate if alternative methods for segmentation would be more relevant for future studies and added this to the discussion section (page 10, lines 336-337). Thank you for this very interesting suggestion. |
| **#54** | -I think would be very helpful to think of ways to consider how these disparities are concentrated. Something like Lorenz curves, Gini index, concentration indices can help to understand how much of the disparities are concentrated at the extremes or whether they are more evenly distributed. Beyond the association, this could help understand where the public health burden really lies. | We fully agree on the relevance of exploring the public health burden in this study. This is also why we chose to report the prevalence proportion differences, as they provide insights into the number of PIMs individuals are subjected to within different social groups. We chose to divide wealth and income into fifths, although investigating the more extreme cases would also be interesting and we will consider this in future studies (please see Table 1). |
| **#55** | -What important measurement issues may be present? These are all hard concepts to actually measure and capture and probably worth some discussion. | Thank you for this suggestion. In the eTable 4 we provide some nuance to the conceptualization of capitals and we reflect on the operationalization of the concepts in the discussion as follows:  “*Nonetheless, social inequality is a complex and entangled issue, and the nuances are difficult to capture fully. Some variables may have acted as mediators or moderators of the association, and the complex nature of the study increased the risk of reverse causation in the study*.” We hope this is sufficient. |
| **#56** | -The use of DAGs in thinking through analyses for these complex phenomena is a strength. I would include these in the supplement so it is clear how each different analysis was conceptualized. This is important work that the authors have done which should be highlighted. | We agree and have added the DAGs to the supplementary to clarify the conceptualization. Thank you for recognizing the importance of this work. |
