# Supplementary material for: Economic, cultural, and social inequalities in potentially inappropriate medication: A nationwide survey- and register-based study in Denmark
Source: PLoS Med. 2024 Nov 20;21(11):e1004473. doi: 10.1371/journal.pmed.1004473 (PMC11578507; doi:10.1371/journal.pmed.1004473)
Supplement: Attachment — Submitted filename: Review response_24_09_28.docx [file pmed.1004473.s010.docx]

**Point-by-point review response**

| **No.** | **Editorial comments** | **Response** |
| --- | --- | --- |
| **#1** | **GENERAL** Thank you for your very detailed and considered responses to previous editor and reviewer comments. Please respond to further comments detailed below prior to publication.  Many of the editorial requests pertain to specific content and formatting requirements, some may not apply and others may have already been incorporated into the manuscript but please review each item and maned as necessary. | Thank you for your detailed feedback. We believe that the revisions have improved the manuscript. Please let us know if any additional changes are required. |
| **#2** | We agree with the reviewer (please see below) regarding the inclusion of the sex disaggregated data within the main manuscript as opposed to supporting information. Please amend accordingly. | We have moved the sex-disaggregated data from supporting information to the main manuscript (figure 3, page 12). |
| **#3** | **DATA AVAILABILITY STATEMENT**  Please include a URL for Statistics Denmark and the Danish Health Data Authority for those wishing to apply for access to data. | We have provided a reference for the webpage with instructions for applying for data access in the data sharing statement uploaded in the manuscript submission form. |
| **#4** | **ABSTRACT**  Please structure your abstract using the PLOS Medicine headings (Background, Methods and Findings, Conclusions).  Please combine the Methods and Findings sections into one section, “Methods and findings”. | We have edited it accordingly. |
| **#5** | Abstract Background: Please provide context of why the study is important. The final sentence should clearly state the study question. | We have added the following sentence to the abstract: “Understanding how social position influences PIM exposure is crucial for improving the targeting of treatment quality and addressing health disparities” |
| **#6** | Abstract Methods and Findings:  Please ensure that all numbers presented in the abstract are present and identical to numbers presented in the main manuscript text. | We have ensured that all numbers in the abstract match those in the main manuscript. |
| **#7** | Please include the study design, population and setting, number of participants, years during which the study took place, length of follow up, and main outcome measures. | The abstract includes study design, population, setting, number of participants, year of investigation, and main outcome measures. It does not include the length of follow-up as the study uses a cross-sectional study design. |
| **#8** | Please quantify the main results with 95% CIs and p values. | 95% CI is included for all relevant results. As for the main manuscript, we have chosen to avoid p-values in this study. Please see response to comment #14 for further clarification. |
| **#9** | Please include the important dependent variables that are adjusted for in the analyses. | These are included (age and sex). Please see page 2 line 47. |
| **#10** | Please include the actual amounts and/or absolute risk(s) of relevant outcomes (including NNT or NNH where appropriate), not just relative risks or correlation coefficients. (example for absolute risks: PMID: 28399126). | We have reported the absolute numbers for PIM exposure in the study population. Please see the section ‘descriptive data’ on page 8. |
| **#11** | Please include a summary of adverse events if these were assessed in the study. | Adverse events were not relevant for this particular study. |
| **#12** | In the last sentence of the Abstract Methods and Findings section, please describe the main limitation(s) of the study's methodology. | We have added a sentence to the study indicating the main limitation. The sentence reads:  “The study's main limitation is the risk of reverse causation due to the complex nature of social position and medical treatment.” |
| **#13** | Abstract Conclusions:  Please address the study implications without overreaching what can be concluded from the data; the phrase "In this study, we observed ..." may be useful.  Please interpret the study based on the results presented in the abstract, emphasizing what is new without overstating your conclusions.  Please avoid vague statements such as "these results have major implications for policy/clinical care".  Mention only specific implications substantiated by the results.  Please avoid assertions of primacy ("We report for the first time....") | We have deleted the following sentence to avoid overreaching and vague statements: “These results suggest that socioeconomic disparities in healthcare persist […], emphasizing the need for targeted interventions to address these inequalities.” |
| **#14** | **STATISTICAL REPORTING**  Throughout, including tables and figures, please quantify the main results with 95% CIs and p values.  When reporting p values please report as <0.001 and where higher as p=0.002, for example. If not reporting p values, for the purpose of transparent data reporting, please clearly state the reasons why not. | We have reported confidence intervals throughout the manuscript but have actively chosen not to report p-values to discourage dichotomous categorizing of the presented results, as suggested by Amrhein and colleagues.  Reference: Amrhein V, Greenland S, McShane B. Scientists rise up against statistical significance. Nature. 2019 Mar;567(7748):305-307. doi: 10.1038/d41586-019-00857-9. PMID: 30894741. |
| **#15** | When reporting 95% CIs please separate upper and lower bounds with commas instead of hyphens as the latter can be confused with reporting of negative values. | We have replaced the hyphens with commas. |
| **#16** | Please include the actual amounts and/or absolute risk(s) of relevant outcomes (including NNT or NNH where appropriate), not just relative risks or correlation coefficients. (example for absolute risks: PMID: 28399126). | Actual amounts (PIM counts), as well as PIM means and standard deviations, are reported separately for all exposure variables. |
| **#17** | **AUTHOR SUMMARY**  Thank you for including an Author Summary which reads very nicely.  The author summary should immediately follow the Abstract in your revised manuscript. Please amend.  It would be helpful to provide a couple of examples of the ‘variables for social position’ that your refer to.  In the final bullet point of ‘What Do These Findings Mean?’, please describe the main limitations of the study in non-technical language. | Thank you. We have inserted the author summary to follow the abstract.  In the fifth bullet point, we now mention all the variables for social position.  We have revised the last bullet points in “What do these findings mean?” so that they now read:  • The study highlights significant social inequalities in PIM exposure, suggesting that socioeconomic disparities in healthcare persist even in a universal healthcare system. Understanding these disparities can guide efforts to reduce inappropriate medication use and improve patient safety  • The findings indicate that economic, cultural, and social capital are crucial determinants of treatment quality, with economic capital showing the strongest association.  • The study's main limitation is the risk of reverse causation due to the complex nature of social position and medical treatment. |
| **#18** | Line 69 – please remove the sub-heading ‘Manuscript’. | This is now removed. |
| **#19** | **INTRODUCTION**  Please ensure that the introduction addresses past research and explain the need for and potential importance of your study. | We have ensured that we covered key studies about potentially inappropriate medication and social inequality in health, and we have also connected those issues to outline the potential importance of our study. We added a sentence on novelty to the final paragraph of the introduction, see reponse to comment #20. |
| **#20** | Indicate whether your study is novel and how you determined that. | We have added a sentence to the introduction on novelty: “To our knowledge, this is the first study to comprehensively explore these associations between social position and PIM.” |
| **#21** | If there has been a systematic review of the evidence related to your study (or you have conducted one), please refer to and reference that review and indicate whether it supports the need for your study. | To our knowledge, no systematic review exists to explore the association between indicators of social position and potentially inappropriate medications. We can add this to the manuscript if needed.  The following search had zero hits: (systematic review[Title/Abstract])  AND ((potentially inappropriate med*[Title/Abstract]) OR (potentially inappropriate pre*[Title/Abstract]) OR (medication us*[Title/Abstract]))  AND ((social inequ*[Title/Abstract]) OR (social position*[Title/Abstract]) OR (social status*[Title/Abstract])) |
| **#22** | **METHODS and RESULTS**  Please report the number of [patients, samples, etc] and dates of recruitment, and account for all methods used in your study. | We have ensured that we reported the number of individuals and relevant dates in the manuscript. Missing data due to non-response is reported in S2. |
| **#23** | Please define "lost to follow-up" as used in this study. Other reasons for exclusion should be defined.  Please define the length of follow up (eg, in mean, SD, and range). | This study is designed as a cross-sectional study, and hence, we had no loss to follow-up. All individuals with complete data across the registries were included in the analyses. |
| **#24** | Please provide the actual numbers of events for the outcomes, not just summary statistics or ORs. | The actual numbers of PIMs are reported in Table 2. |
| **#25** | Please present numerators and denominators used to derive percentages. | The percentages and mean number of PIMs presented in the results section is found in table 2 and 3. Exposure to START and STOPP PIMs have been clarified in the main text as these numbers do not appear directly in the table:  “Exposure to START PIMs (22,140/177,495=12.5%) was more common than STOPP PIMs (5,555/177,495=3.1%).” Please see the section ‘descriptive data’ in the results section. |
| **#26** | Please ensure to indicate where analyses are adjusted and which factors are adjusted for. | The description of each model now specifies that it was adjusted for age and sex. For model 2 and 3 we also indicate how the analysis was stratified. |
| **#27** | As for the abstract, please ensure to quantify the main results with 95% CIs and p values.  When a p value is given, please specify the statistical test used to determine it. | As described in #14, we have reported confidence intervals throughout the manuscript but have chosen not to report p-values to discourage dichotomous categorizing of the presented results, as suggested by Amrhein and colleagues. |
| **#28** | As above please include the disaggregated data within the main manuscript | We have now included the sex-stratified analyses in the main manuscript. |
| **#29** | **TABLES and FIGURES**  As above please include the disaggregated data within the main manuscript.  Please see here for guidelines on submitting and citing figures ([link](https://journals.plos.org/plosmedicine/s/figures#loc-how-to-submit-figures-and-captions))  Please provide titles and legends for all tables and figures (including those in Supporting Information files).  Please ensure that each table and figure is affiliated to a caption which clearly describes the figure content without the need to refer to the text.  Please ensure that all abbreviations are defined in the caption or an appropriate footnote, including those used to report statistical information.  Please ensure to include the meaning of any dots/lines/bars.  Please consider avoiding the use of red and green in order to make figures more accessible to those with colour blindness.  To help facilitate transparent data reporting, where adjusted analyses are presented please also present the unadjusted analyses for comparison. In a caption or footnote please detail all factors adjusted for. | We have included the sex-stratified data within the main manuscript (figure 3), provided titles and legends for all tables and figures, defined all abbreviations and meanings of dots/lines/bars, avoided red and green colors, and detailed factors adjusted for in the legend. |
| **#30** | **DISCUSSION**  Please ensure to present and organize the Discussion as follows: a short, clear summary of the article's findings; what the study adds to existing research and where and why the results may differ from previous research; strengths and limitations of the study; implications and next steps for research, clinical practice, and/or public policy; one-paragraph conclusion. Please avoid the use of sub-headings such that the discussion reads as continuous prose. | We have organized the discussion section as a summary of findings, contributions to existing research, strengths and limitations, implications, future research, and a one-paragraph conclusion. |
| **#31** | Lines 347, 365 and 367 – please remove these statements from the main manuscript and include only in the manuscript submission form when you resubmit your manuscript. At the time of publication these will be compiled as metadata. | We have removed the following statements for inclusion in the manuscript submission form:   - Data Sharing Statement - Conflicts of Interest - Role of funding sources |
| **#32** | **REFERENCES**  For in-text reference callouts please place citations in square parentheses separate by commas. For example, [1,3,6] or [1-3]. Please check and amend throughout all sub-sections of the manuscript and supporting files.  In the bibliography please ensure that you list up to but no more than 6 author names followed by et al.  For all web references please ensure you include an, ‘Accessed [date].’  Journal name abbreviations should be those listed in the National Center for Biotechnology Information (NCBI) databases. | We have ensured in-text references are in square parentheses, limited to 6 author names in the bibliography followed by et al., and used NCBI-listed journal abbreviations. Access dates for the one web references included in meta data. |
| **#33** | **SUPPORTING INFORMATION**  In the published article, supporting information files are accessed only through a hyperlink attached to the captions. For this reason, you must list captions at the end of your manuscript file. You may include a caption within the supporting information file itself, as long as that caption is also provided in the manuscript file. Do not submit a separate caption file.  As the supporting information files are contained with a single file, please label and cite the ‘supplementary appendices’ and ‘initial project description’ as follows:  Please label the files as ‘S1 Supplementary appendices’ and ‘S2 Study protocol and analysis plan’ respectively.  Please apply alphabetical labelling to each table and figure contained within the S1 file. For example, ‘Fig A’ to ‘Fig Z’ and ‘Table A’ to ‘Table Z’.  Plain text does not need to be labelled and can just be given a title as necessary. For example, ‘Statistical Analysis Plan’.  Please cite tables/figures as ‘Fig A in S1 Supplementary appendices’ and/or ‘Table A in S1 Supplementary appendices’, for example. | After conclusion, we have inserted a section entitled Supporting Information.  As described in our last review response, we do not think the initial protocol is fit for publication but only uploaded it for the reviewers’ reference. We have divided the remaining appendices in separate files to provide a better overview for readers.    The captions now read: S1 Table. Response rates in the Danish National Health Survey 2017  S2 Table. Missing data  S3 Table. PIM criteria definitions according to the register-adapted STOPP/START criteria  S4 Table. Description of operationalization of capital forms  S5 Table. Long-term conditions in the Danish Multimorbidity Index  S6 Figure. Illustration of variables with Directed Acyclic Graph (DAG) |
| **#34** | **SOCIAL MEDIA**  To help us extend the reach of your research, please detail any X (formerly Twitter) handles you wish to be included when we tweet this paper (including your own, your coauthors’, your institution, funder, or lab) in the manuscript submission form when you re-submit the manuscript. | We have included the relevant information to include us in social media. |

|  | **Comments from Reviewers:** |  |
| --- | --- | --- |
| **#35** | **Reviewer #1:** The authors have addressed all my points | Thank you once again, we appreciate the effort. |
| **#36** | **Reviewer #2:** Thank you for your revised manuscript. You have done a great job of meeting all reviewers' comments and the manuscript has now improved.  However, I do have a continuing remark regarding gender stratification:  I appreciate that the appendix now have separate tables and figures for women and men and, to stress my point, there are differences. Not many, but enough to mention in the results and discussion.  I agree that it is a lot of data but I would have preferred to have the gender stratified tables and figures in the main article, not in the appendix, but I leave this decision to the editor. | We appreciate the suggestion and have included the gender-stratified tables and figures in the main article, as requested. Thank you for your effort in reviewing our work. |
| **#37** | Also, maybe the interaction analysis would be too much for the main article, but since you have performed the analysis, it would have been nice to see the results (gender stratified!) in the appendix. | After thorough discussions among the authors, we have decided not to include the interaction analysis in the appendix due to the overwhelming amount of data. |
| **#38** | Finally, eFigure 2 clarifies the difference between models a and b in the heading (START and STOP). This explanation would benefit the figures in the manuscript. | Thank you for the suggestion. We have clarified the headings in the remaining figures in the manuscript. |
| **#39** | **Reviewer #3:** Thank you for the opportunity to review this manuscript again. The authors have done a great job revising the analytical and theoretical part of the manuscript. I have some minor comments that I think would improve an already well-developed manuscript.  Below, I list some issues that in my view, would improve the paper. | Thank you for your comments, we appreciate your effort in reviewing the manuscript. |
| **#40** | Minor comments:  1. The separation of PIMS into STOP and START have improved the paper substantially, I now agree with the analytical decisions. However, the authors should revise how they describe these results in the Result-section and in Discussion (under the heading key-finding) for style and clarity. | We have worked to improve the style and clarity of the results sections and the initial summary in the discussion. |
| **#41** | 2. Row 327-329. I would argue that both gastrointestinal and cardiovascular complications are more recognized side-effects of long-term NSAID use in older adults. Consider changing. | We have edited it accordingly. |
